# Comparison of the Immune Enhancing Activity and Chemical Constituents Between Imitation Wild and Cultivated Astragali Radix

**DOI:** 10.3390/molecules30040923

**Published:** 2025-02-17

**Authors:** Shuo Zhao, Xueting Li, Yumeng Wang, Rui Xu, Xu Li, Jiushi Liu, Xiaolin Hou, Haitao Liu

**Affiliations:** 1 Animal Science and Technology College, Beijing University of Agriculture, Beijing 102206, China; zsdwyyx0918@163.com (S.Z.); wym3684@163.com (Y.W.); lixu9806@163.com (X.L.); 2 State Key Laboratory for Quality Ensurance and Sustainable Use of Dao-Di Herbs, Institute of Medicinal Plant Development, Chinese Academy of Medical Sciences and Peking Union Medical College, Beijing 100193, China; lxt1041233448@163.com (X.L.); 13523935177@163.com (R.X.)

**Keywords:** imitation wild Astragali Radix, cultivated Astragali Radix, combination therapy, breast cancer, UPLC-Q-TOF/MS

## Abstract

Astragali Radix (AR), a traditional food and medicinal herb used for thousands of years, is widely recognized for its role in enhancing immunity, particularly when combined with adjuvant chemotherapy. The two primary types of AR available in the market are imitation wild AR (grown for seven years) and cultivated AR (grown for two years). However, whether differences exist in their immune-enhancing effects and chemical constituents remains unclear. In this study, a comparative analysis was performed to evaluate the immune activity and chemical composition of cultivated and imitation wild AR. Immune activity was assessed through in vivo animal studies, while metabolomic analysis was used to characterize their chemical profiles. The results demonstrate that AR possesses significant antitumor and immune-enhancing activities, with imitation wild AR showing superior efficacy compared with cultivated AR. Following 16 days of daily AR treatment, both the thymus and spleen coefficients were significantly increased, effectively reversing the immune dysfunction induced by cyclophosphamide (CTX). Moreover, the administration of AR showed no significant toxicity, as evidenced by the stable liver and kidney function indicators, including ALT, UREA, and CRE levels. To investigate chemical differences, a customized chemotaxonomic-based in-house library containing 215 compounds was developed and integrated with the Progenesis QI informatics platform for metabolite annotation. Using multivariate analysis, 48 constituents were identified in total: 46 unique to the imitation wild AR and 45 specific to the cultivated AR. The correlation between chemical constituents and the pharmacological effects of AR was evaluated. Based on orthogonal partial least-squares discriminant analysis (OPLS-DA) and S-plot analysis, five potential biomarkers were identified, including Calycosin-7-glucoside, Rhamnocitrin-3-O-β-D-glucopyranoside, Ononin, 3,5-Dicaffeoylquinic acid, and Acetylastragaloside I. These biomarkers likely account for the differences in immune-enhancing effects between the two AR types. This study provides a scientific foundation for the rational use of Astragali Radix.

## 1. Introduction

Astragali Radix (AR, also known as Huang Qi in China) is an important food and medicine plant that has been used in China for thousands of years and was first recorded in the Shennong Bencao Jing [1]. AR is famous for its ability to enhance immunity [2,3]. AR is mainly produced in the Northwest and Northeast of China. The main components of AR include flavonoids, saponins, polysaccharides, amino acids, and trace elements [4]. Many studies have shown that AR can enhance human immunity and effectively treat various cancers, cardiovascular diseases, hypertension, and diabetes [5,6]. In 2023, AR was included in the list of food and traditional Chinese medicine substances by the National Health Commission. It is commonly consumed in soups and is also listed as a traditional Chinese medicine in the Chinese Pharmacopoeia [7].

With the increasing demand for AR, imitation wild AR and cultivated AR are the mainstream sources of the commodity [8]. Imitation wild AR is mainly produced in Hunyuan County, Shanxi Province, which is an arid mountainous area with a minimum growth period of 7 years [9]. Cultivated AR is usually cultivated on flat land, sown, and nurtured for one year, then transplanted and grown for another year before harvesting [10]. In the process of market sales and use, the commodity grade specifications and prices of imitation wild AR and cultivated AR are different [11]. Studies have revealed that wild-simulated Astragalus exhibits superior therapeutic efficacy over its cultivated counterpart in treating heart failure in murine models [12,13]. Nonetheless, the relationship between the inherent quality of medicinal materials across various grades and their immunotherapeutic effectiveness is still not well understood. Therefore, it is very important to evaluate and compare the chemical composition and immune effects of imitation wild and cultivated AR.

Breast cancer is a common malignant tumor that seriously endangers women’s health [14,15]. Cyclophosphamide is widely used as a chemotherapy drug in clinical practice [16]. Due to its immunosuppressive and cytotoxic effects, AR is often used in combination with it [17,18,19,20]. The combination therapy of AR can reduce the adverse reactions of chemotherapy and improve the immune function of cancer patients [21,22,23]. However, the immunomodulatory effects of imitation wild AR and cultivated AR extracts are still unclear. In this study, a mouse model of breast cancer was established, and the effects of AR extracts from different cultivation methods combined with cyclophosphamide chemotherapy were evaluated. In addition, metabolomics analysis was conducted on the chemical components of imitation wild AR and cultivated AR to identify potential differentially active compounds. We hope that our research can provide a basis for the clinical application of AR.

## 2. Result and Discussion

### 2.1. Result

#### 2.1.1. The Synergistic Chemotherapy Effect of Imitation Wild and Cultivated Astragali Radix

**Figure 1:**
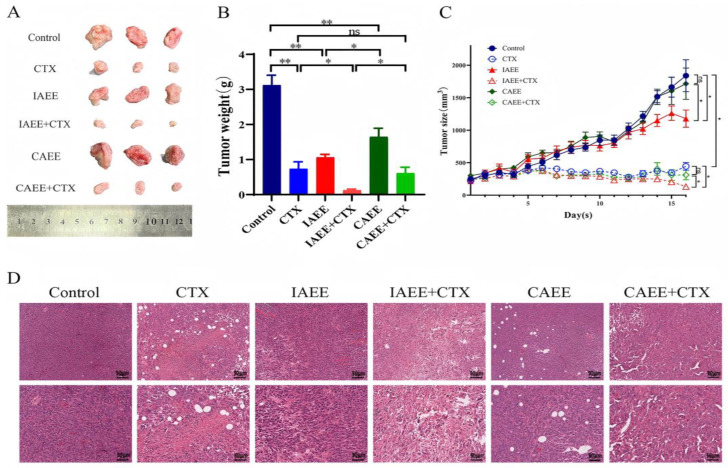
The antitumor effect of single and combined therapy of Astragali Radix from different sources: (**A**) Schematic diagram of tumor size after administration in each group. (**B**) Changes in tumor mass after administration. (**C**) Changes in tumor volume during the experiment; (**D**) Schematic diagram of H&E staining of tumor tissue, scale bar = 50 μm; The results are expressed as mean ± SEM, * *p* < 0.05, ** *p* < 0.01, ns: no significant difference (n = 6).

In this study, a mouse breast cancer model was established by subcutaneous inoculation of 4T1 cells in Balb/c mice, and CTX was selected as the chemotherapeutic agent. In order to investigate the synergistic effect of CTX combination therapy on breast cancer, the imitation wild AR ethanolic extract (IAEE) and cultivated AR ethanolic extract groups (CAEE) were set up for single therapy as well as IAEE+CTX and CAEE+CTX combination therapy groups. As shown in Figure 1B,C, the size and weight of the tumor have significantly decreased. But the IAEE and combined CTX groups were significantly better than CAEE. This result is also clearly visible in appearance. As shown in Figure 1B,C, compared with the control group, the tumors in the CTX group, the IAEE and CAEE groups, and the IAEE+CTX and CAEE+CTX groups were significantly reduced, and the IAEE+CTX group was significantly different from the CTX group as well as the CAEE+CTX group in terms of tumor size and weight. In addition, the immune-enhancing activity after treatment with CTX and both AR ethanolic extract combined with CTX treatment was also clearly visible from the appearance of the tumors (Figure 1A).

The pathological examination of tumor tissues by H&E staining was performed to further evaluate the synergistic chemotherapy effect of both AR ethanolic extract (Figure 1D H&E staining of tumor tissues). The tumor tissues in the control group were intact and clear, and the tumor cells had abnormally enlarged nuclei, dense chromatin and partly binucleated, irregular cell arrangement, obvious cell heterogeneity, elevated nucleoplasm ratio close to 1:1, and strong cytoplasm basophilia. After CTX treatment, the tumor cells had significantly reduced nuclei, loose cell arrangement, and nuclear fragmentation, indicating a large number of tumor cell necrosis. Tumor cells in the IAEE group showed some nuclei reduction, cell shrinkage, and fragmentation. After the combination of IAEE and CTX treatment, tumor cells showed a significant reduction in nuclei, more pronounced cell shrinkage and fragmentation, and the cell pleomorphism decreased significantly.

These results indicate that the ethanolic extract of AR has a synergistic effect on the antitumor activity of CTX, which can enhance the chemotherapeutic effect of CTX. In addition, the tumor suppression effect of IAEE and IAEE+CTX combination therapy was more significant than that of CAEE group and CAEE+CTX combination therapy group.

#### 2.1.2. Immune Enhancement Effect of Imitation Wild and Cultivated Astragali Radix

**Figure 2:**
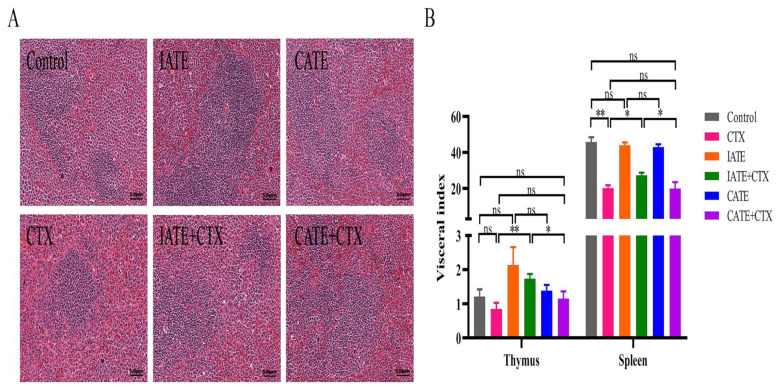
Effects of different cultivation methods of Astragali Radix and combined treatment on immune regulation of Astragali Radix: (**A**) Diagram of spleen tissue H&E staining in each group after administration, scale bar = 50 μm. (**B**) The effect of drug administration on the organ coefficient of mice. The results are expressed as mean ± SEM, * *p* < 0.05, ** *p* < 0.01, ns: no significant difference (n = 6).

Changes in the weight of the thymus and spleen can reflect whether an immune response has been triggered in the body. Thus, we used this indicator to evaluate the immunostimulatory effects of Astragalus under different cultivation methods. As shown in Figure 2, although cyclophosphamide (CTX) only partially impaired thymic immune function, co-administration with IAEE significantly increased the thymus index. According to changes in the spleen index and H&E staining results (Figure 2A,B), CTX treatment led to a blurred boundary between the red and white pulp in the spleen and a severe reduction in the proportion of white pulp, indicating a decrease in lymphocyte proportion and a substantial impact on spleen immune function. However, administration of IAEE alleviated this phenomenon, as the spleen index increased. IAEE significantly reversed the CTX-induced decline in immune function, with a stronger effect than CAEE.

#### 2.1.3. Effects of Imitation Wild and Cultivated Astragali Radix on the Levels of Enzymes and Factors Related to Immune Enhancement in Tumors Tissue of Mice

**Figure 3:**
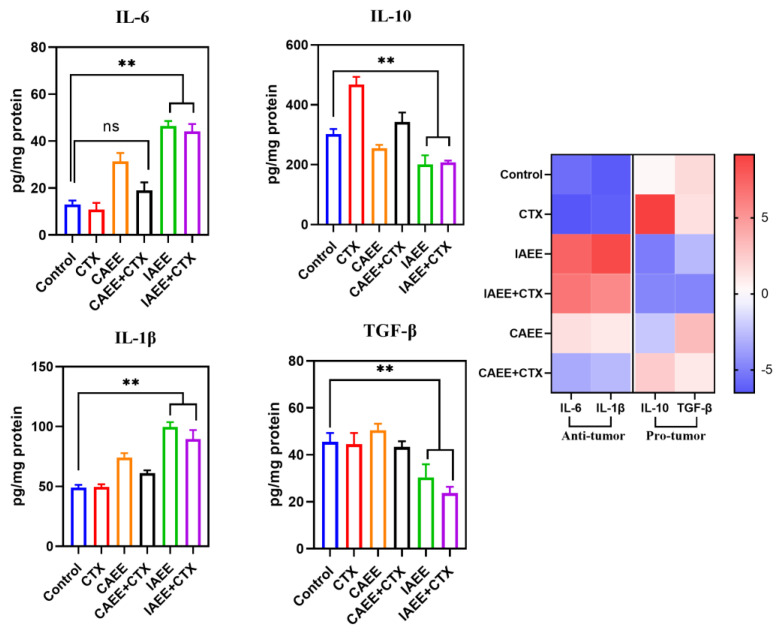
Effects on tumor tissue inflammatory cytokines in mice, ** *p* < 0.01, ns: no significant difference (n = 6).

Immunological response represents a critical pathological process in tumor development, predominantly characterized by the abnormal secretion of cytokines. ELISA analysis demonstrated significant differences in the levels of four cytokines between the control and model groups. Compared with the model group, all experimental groups exhibited a significant increase in IL-6 and IL-1β levels, alongside a reduction in IL-10 and TGF-β levels. Notably, the imitation wild group (IAEE) showed higher levels of IL-6 and IL-1β and lower levels of IL-10 and TGF-β compared with the cultivated group (CAEE) (Figure 3).

Interleukin-6 (IL-6) is markedly upregulated in the tumor microenvironment, promoting tumor progression and suppressing antitumor immune responses via activation of the JAK/STAT3 signaling pathway [24]. Similarly, IL-1β, an immune amplification signal, participates in the progression of cancer and inflammatory diseases, with evidence indicating its tumor-promoting role in various malignancies [25]. The results suggest that the imitation wild group exhibits superior immune-enhancing effects compared with the cultivated group. Interleukin-10 (IL-10) is a critical immunoregulatory factor with immunosuppressive functions. It contributes to the establishment of a local immune-suppressive tumor microenvironment by restricting both adaptive and innate immune responses, thereby significantly impairing the ability of the host’s immune system to eliminate tumor cells [26]. Similarly, transforming growth factor-beta (TGF-β) plays a key inhibitory role in the development, activation, and differentiation of lymphocytes in cancer, promoting immune evasion and tumor progression [27]. The results indicate that the pseudo-wild group is capable of activating and enhancing immune responses, thereby improving antitumor efficacy. Notably, IL-10 levels were significantly elevated in mice following chemotherapy, suggesting that chemotherapy may exacerbate the tumor’s immune environment. However, this phenomenon was markedly improved when combined with the treatment, highlighting the beneficial effects of the pseudo-wild group in not only improving the tumor immune microenvironment but also mitigating the immune-related side effects associated with chemotherapy.

#### 2.1.4. Evaluation of Drug Effects on Major Organ Tissues

**Figure 4:**
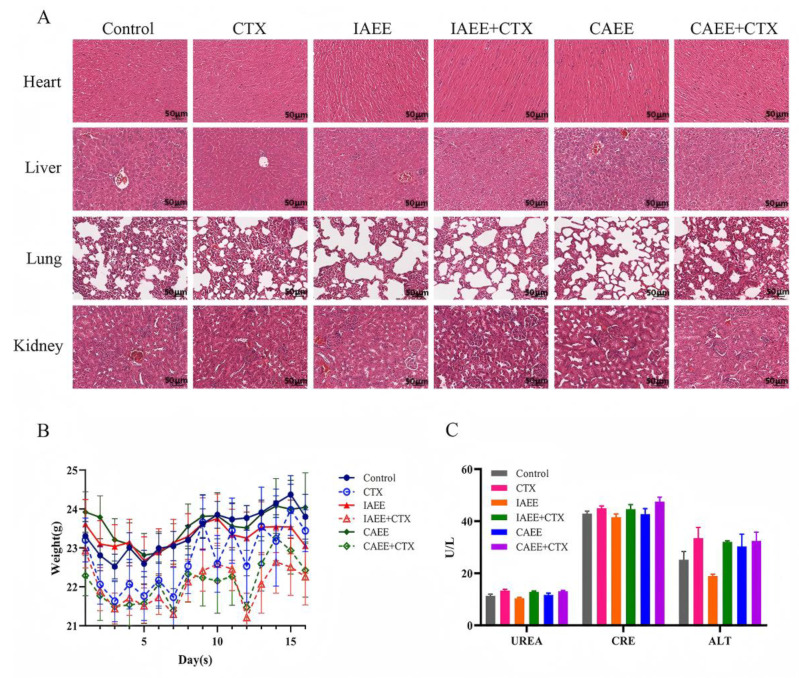
Effects on major organs after administration: (**A**) Schematic diagram of H&E staining of major organs in each group after drug treatment, scale bar = 50 μm. (**B**) Line chart of body weight changes in mice. (**C**) Statistical chart of UREA, CRE, and ALT content in serum. Results are expressed as the mean ± SEM (n = 6).

CTX, a commonly used alkylating antitumor agent, is converted in the liver to produce alkylation in vivo, thus inhibiting tumor cell proliferation [28,29]. However, CTX can damage normal cells, suppress immune function, and potentially induce additional diseases [30,31,32]. Thus, evaluating its potential in vivo toxicity is essential. Throughout the experimental period, no significant weight changes were observed in any treatment group compared with the control group (Figure 4B). Moreover, H&E staining results show no significant histopathological damage to the organs in any treatment group (Figure 4A). Liver and kidney function were further evaluated by blood biochemical tests at the end of the experiment. Elevated ALT (alanine transferase) indicates acute hepatocellular injury, while elevated UREA and CRE (creatinine) serve as markers of renal function damage. As shown in Figure 4C, serum levels of ALT, UREA, and CRE in treated mice did not significantly differ from the control group, with all serum biochemical indices remaining within healthy ranges. This suggests that the selected chemotherapeutic agents and the administered dose of AR ethanolic extract had no significant effects on liver or kidney function in mice during this experiment.

#### 2.1.5. Construction of the Astragalus Compound Reference Local Library

One of the key challenges in untargeted metabolomics research is identifying metabolites from complex matrices, especially bioactive molecules. In the absence of standard compounds, internal libraries serve as valuable tools for biomarker annotation, as they offer higher accuracy and success rates compared with untargeted public and commercial databases, such as the NIST library, that are directly used for metabolite characterization in LC-MS metabolomics analysis. The chemical composition of Astragalus has been extensively documented in the scientific literature. We collected all published articles on phytochemical research in the last 12 years from 2010 to 2022 by searching electronic databases such as PubMed, Web of Science, Reaxys, Google Scholar, SciFinder, and CNKI for keywords related to Astragali Radix and performed an exhaustive and systematic compilation of the compounds reported in the literature. Therefore, a total of 215 metabolites are included in the established local database, including flavonoids, triterpenoids, saponins, organic acids, and alkaloids, among others. This customized library can be used for metabolite characterizations for Astragali Radix, particularly for the varying growth durations in nontarget metabolomic analysis.

#### 2.1.6. MS Data Processing and Metabolite Characterization in Astragali Radix

**Figure 5:**
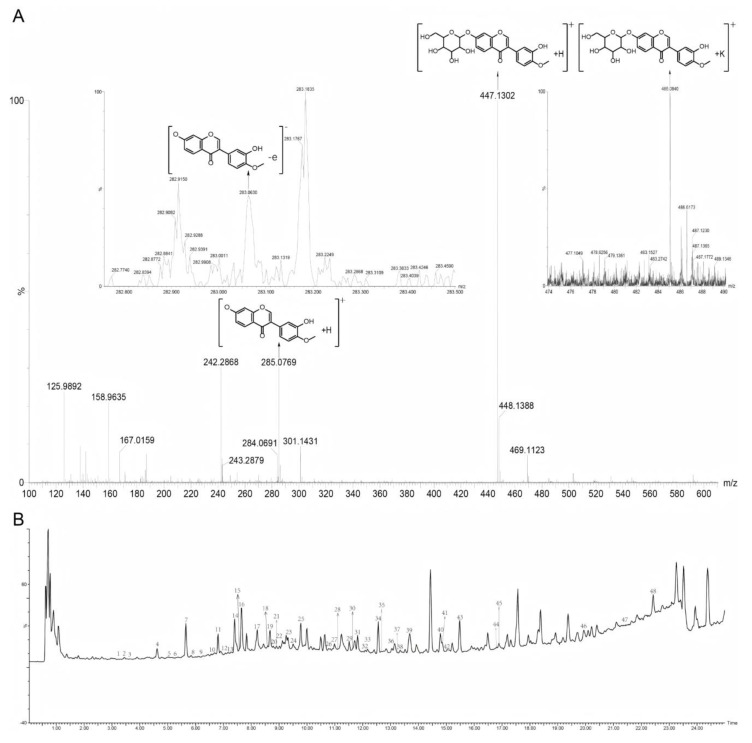
The identification process of chemical components in positive ion mode and TIC mass spectrometry of chemical components in Astragalus membranaceus: (**A**) Identification spectrum of Calycosin-7-glucoside. (**B**) Chemical composition TIC mass spectrum of Astragalus membranaceus in positive ion mode. 1. Rutin; 2. 3′,7,8-trihydroxy-4′-methoxyisoflavone; 3. Chrysoeriol-7-O-D-glucopyranosyl-4′-O-α-L-rhamnopyranoside; 4. Syringic acid; 5. Quercetin 3-O-β-D-glucopyranoside; 6. Dihydroxy-6-methoxyaurone; 7. Calycosin-7-glucoside; 8. Verbascoside; 9. 2′-Hydroxy-di-methoxyisoflavan-β-D-glucoside; 10. Liquiritigenin; 11. 8,2′-Dihydroxy-7,4′-dimethoxyisoflavan; 12. Vanillin; 13. 7-Hydroxy-2′, 3′, 4′, 5′-tetramethoxyisoflavan; 14. Astraflavonoid B; 15. Rhamnocitrin-3-O-β-D-glucopyranoside; 16. Oroxylin A; 17. Ononin; 18. Homobutein; 19. 9-O-Methylnissolin 3-O-glucoside; 20. Oxytropisoflavan B; 21. Astragaloside VIII methyl ester; 22. Rhamnocitrin; 23. Astroolesaponins F; 24. Astragaloside II; 25. 3,5-Dicaffeoylquinic acid; 26. Betulinic acid; 27. Astragaloside III; 28. Astroolesaponins B; 29. Astragaloside Ⅳ; 30. Robinioside B; 31. Robinioside F; 32. Chrysoeriol-4′-O-α-L-rhamnopyranoside; 33. Astraisoolesaponins A1; 34. Iso-astragaloside II; 35. Cloversaponin III; 36. Cyclocephaloside II; 37. Kaempferol-4′-methyl ether; 38. AstragalosideI; 39. IsoastragalosideI; 40. Cycloastragenol; 41. 2′,4,4′-trihydroxychalcone; 42. Astroolesaponins A; 43. Astroolesaponins D; 44. Astragaisoflavan D; 45. AcetylastragalosidI; 46. Methylnissolin; 47. Swainsonine; 48. Stigmasten-4-en-3-one.

After peak picking and deconvolution by Progenesis QI, a total of 9825 feature ions were generated, suggesting a high chemical complexity in Astragali Radix. The approach for the tentative identification of a single metabolite in Progenesis QI is presented in Figure 5A, taking calycosin-7-glucoside as an example. After deconvolution, two adducts, [M+H]^+^ = 447.1286 *m*/*z* and [M+K]^+^ = 485.0840 *m*/*z* at 5.69 min, and the mass error and isotope similarity score were determined (Figure 5A). In addition, the typical fragment ions (283.0624 *m*/*z*, C16H11O5-e; 285.0758 *m*/*z*, C16H12O5+H) from the high-energy spectrum were further assigned by the informatics platform, providing a fragmentation score (Figure 5A). Based on mass error, isotope similarity, and ion assignment, the feature was annotated as calycosin-7-glucoside with a “Score” of 51.3 in Progenesis QI (Figure 5A). This tentative identification was further verified by comparisons of the retention time and low- and high-energy MS data between the sample with the detected feature and the standard compound in co-injection experiments, indicating the reliable identification by Progenesis QI using the Astragali Radix in the local library. For another, at 9.52 min and 12.56 min, two compounds were detected with a molecular weight and adduct ion [M+Na]^+^ = 849.4634 *m*/*z*, tentatively identified as tetracyclic triterpenoid saponins, specifically astragaloside II and isoastragaloside II. As is well-known, astragaloside II exhibits greater polarity than isoastragaloside II. Furthermore, saponins generally undergo neutral losses, resulting in fragment losses such as -Glu, -GluA, -Rha, and -Ara. Based on typical fragment ions such as C7H9O4-e (157.0527 *m*/*z*), C31H48O8+Na (575.3245 *m*/*z*), C37H57O8-e (629.4131 *m*/*z*), and C30H59O9-e (647.4231 *m*/*z*), the compound at 9.52 min was identified as astragaloside II, and by analogy, the compound at 12.56 min was inferred to be isoastragaloside II (Figure 5B). As a result, a total of 48 compounds were annotated from the imitation wild AR (5-year) and cultivated AR, including 29 flavonoids and their glycosides, 16 terpenes and their glycosides, 2 organic acids, and 1 nitrogen-containing compound or alkaloid. Detailed information on the characterized chemicals, including adducts, molecular formula, score, and identification, are presented in Table 1.

#### 2.1.7. Metabolite Differences and Biomarker Characterization Studies of Imitation Wild and Cultivated Astragali Radix

**Figure 6:**
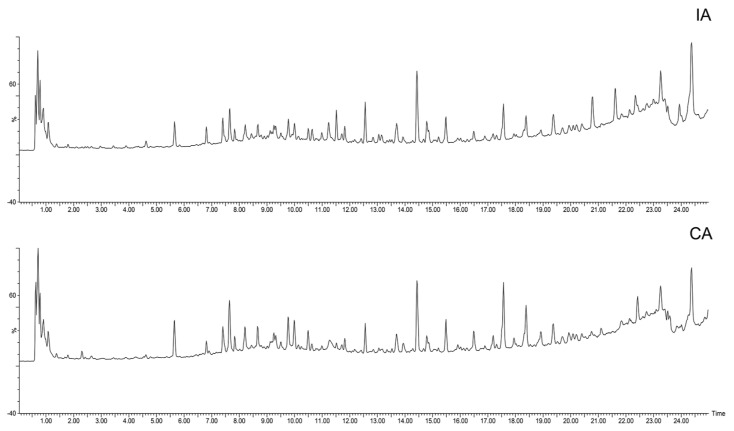
Comparison of total ion flow graphs (TIC) of IA and CA.

**Figure 7:**
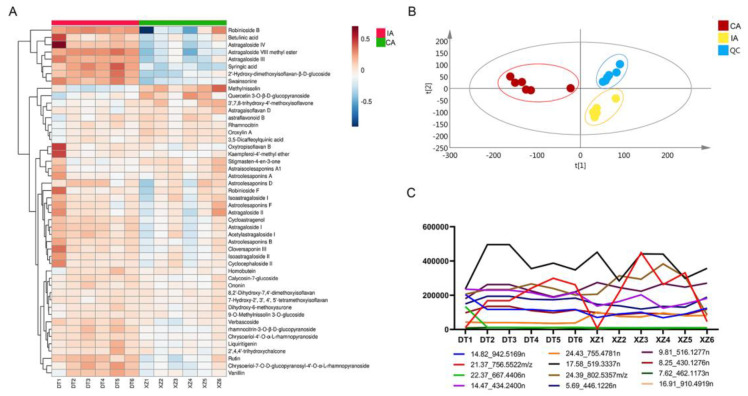
Schematic representation of the chemical composition differences between IA and CA: (**A**) A heatmap based on the relative abundance of 48 identified compounds in different parts of IA and CA. (**B**) A PCA score plot of three parts of Astragali Radix: imitation wild Astragali Radix (IA), cultivated Astragali Radix (CA), and quality control (QC). (**C**) Examples of variation trends of different compounds in different parts of Astragali Radix.

The total ion flow diagrams (TIC) of the imitation wild and cultivated AR (IA and CA) (Figure 6) showed significant differences in their overall composition and some compounds were not identified in IA or CA, such as Syringic acid, 2′-Hydroxy-dimethoxyisoflavan-β-D-glucoside, astragaloside VIII methyl ester, Astroolesaponins F and Astroolesaponins D (Figure 5 and Table 1). To visualize the metabolite differences among samples and further uncover the highly discriminative compounds, PCA analyses were conducted. In order to understand the distribution characteristics of the identified compounds in different sources of AR, we used a heat map as a clearer and more visual way to visualize the data. It is noteworthy that all peaks identified in this study in the heat map were significantly different in different cultivation methods of AR (Figure 7A). In the PCA models for imitation wild AR and cultivated AR, PC1 and PC2 explained variances of 30.2 and 18.8% and 23.4 and 17.9%, respectively. According to the results of the cluster analysis in the positive ion mode, it is clear that the QC sample points are better aggregated, which indicates better stability and precision of the instrument, and under these conditions, there is a certain trend of separation between the two different sources of AR with significant diversity between groups (Figure 7B), which is consistent with the results shown in the TIC plots, where the compounds have more different peaks in different sources of AR (Figure 7A,C), for example, Isoastragaloside II IAEE > CAEE and Rutin IAEE > CAEE.

**Figure 8:**
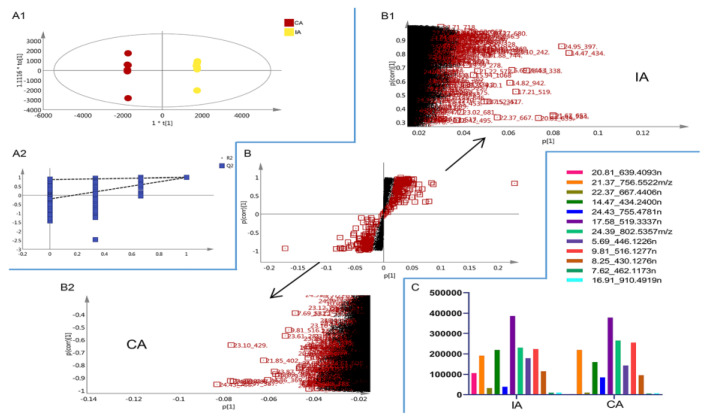
An example of marker compound selection in Astragali Radix: (**A**) orthogonal partial least-squares discriminant analysis (OPLS-DA) score plot (**A1**) and the reliability of the OPLS-DA model (**A2**). (**B**) S-plot score of (**B1**) IA and (**B2**) CA. (**C**) The trend of the relative contents of different compounds in IA and CA.

The S-plot affiliated with the OPLS-DA model is a useful strategy to visualize the metabolic differences between two groups and to further characterize potentially bioactive constituents from the group with stronger bioactivity. The significant variables that are considered as potential biomarkers associated with biological activity are plotted at the upper or lower ends of the S-plot, while the variances that do not show significant contribution are located in the middle. OPLS-DA models and their corresponding S-plots were established, and the evaluation parameters R^2^ = 1 and Q^2^ = 0.992 of the OPLS-DA model indicate that the model is more stable in this experiment, in which the mean value of both is greater than 0.9 (Figure 8A). Based on the OPLS-DA model showed that the biomarker molecules were highlighted in the S-plots based on the selection criteria: VIP > 3, |*p*| > 0.05, and |*p*(corr)| > 0.5 (Figure 8B). The x-axis in the S-plot is the covariance coefficient between the main component and the metabolite, and the y-axis is the correlation coefficient between the main component and the metabolite, so the further away from the axes the data are, the stronger the variance is, and the compounds with stronger variance are different in the two AR groups (Figure 8C). We selected compounds with VIP > 3 and *p*-value < 0.05 as the differential chemical components with significant differences. It was found that Calycosin-7-glucoside, Rhamnocitrin-3-O-β-D-glucopyranoside, Ononin, 3,5-Dicaffeoylquinic acid, and Acetylastragaloside I compounds that have been identified in this experiment showed significant differences, respectively (Table 2), and most of these compounds are the main active compounds in Astragalus, among which flavonoids have been reported as a class of compounds with immune-enhancing activity in AR.

### 2.2. Discussion

Astragali Radix is a dual-purpose substance used in both food and medicine, renowned for its efficacy in replenishing vital energy (Qi) and promoting Yang energy. Cancer patients often co-administer Astragali Radix with cyclophosphamide, as Astragali Radix has been shown to enhance immune function.

Astragali Radix (AR) is a dual-purpose substance valued for both its culinary and medicinal applications, renowned for replenishing vital energy (Qi) and promoting Yang energy. Cancer patients often co-administer AR with cyclophosphamide (CTX) due to its immune-enhancing properties. Previous studies have identified key components of AR, such as flavonoids and saponins, which align with our findings. These compounds have demonstrated significant immune-enhancing effects and the ability to enhance the sensitivity of chemotherapeutic drugs. For instance, astragalosides have been shown to stimulate the proliferation of T- and B-lymphocytes in splenocytes via ConA and LPS, respectively, and boost T-lymphocyte proliferation in response to anti-CD3 stimulation [33,34]. Studies have also reported that astragalosides I, II, III, and IV exhibit substantial IL-2-inducing activity in whole blood samples stimulated by phytohemagglutinin, contributing to their immunostimulatory and anticancer effects [35]. Additionally, polysaccharides in AR enhance the inhibitory effects of chemotherapeutic drugs on tumor cells, mitigate immune disorders, and increase drug sensitivity [36].

Imitation wild AR commands a significantly higher market price than cultivated AR, based on the perception that pseudo-wild AR exhibits superior immune-enhancing properties. However, this assertion lacks scientific validation. In our study, we compared the efficacy of two AR sources using a mouse model of breast cancer. Our findings revealed that treatment with AR total extract significantly reduced tumor mass and volume compared with the untreated group. Histological analysis (H&E staining) of tumor tissues demonstrated substantial tumor cell nuclear reduction, fragmentation, and decreased anisotropy in the AR-treated group. These changes paralleled those observed in the CTX-treated group. Notably, the combined administration of AR and CTX resulted in marked reductions in tumor cell size, increased fragmentation, and enhanced chemotherapy efficacy. The immune-enhancing activity of imitation wild AR was found to be superior to that of cultivated AR.

To further explore differences in active biomarkers, we conducted a metabolomics analysis of the chemical composition of imitation wild and cultivated AR. Five potential biomarkers were identified, including flavonoids, isoflavonoids, saponins, and organic acids, which are the primary active compounds in AR with immunomodulatory, antitumor, and antioxidant properties. Flavonoids, such as calycosin-7-glucoside, exhibit potent anti-inflammatory effects by reducing the secretion of inflammatory mediators (e.g., NO, IL-1β, IL-6, IL-12, TNF-α) and downregulating the expression of CD86 and iNOS. This compound also promotes anti-inflammatory cytokines (e.g., IL-10, VEGF) and enhances M2 macrophage markers (e.g., CD206, Arg-1) [37]. Ononin achieves these effects by inhibiting COX-2 and iNOS expression and modulating the NF-κB and MAPK signaling pathways. Organic acids in AR, such as 3,5-dicaffeoylquinic acid, also exhibit immune-enhancing effects by attenuating LPS-induced microglial activation and pro-inflammatory cytokine production [38,39]. The identification of these biomarkers provides a scientific basis for the authentication and quality control of imitation wild AR.

## 3. Materials and Methods

### 3.1. Materials

The imitation wild AR (5-year) and transplanted AR (2-year) were collected in October 2023 from Hunyuan, Datong, and Wuzhai, Xinzhou, both located in Shanxi Province, China, respectively. Both herbs were authenticated by Prof. Yaodong Qi, a researcher from the Institute of Medicinal Plant Development (IMPLAD) in Beijing, China. Subsequently, they were preserved in the Herbarium of the Institute of Medicinal Plant Development.

### 3.2. Chemicals and Reagents

The analytical-grade alcohol was procured from Beijing Chemical Works (Beijing, China). Pure water (18.2 MΩ) was obtained from a Milli-Q System (Millipore, Billerica, MA, USA). Cyclophosphamide (CTX) for injection was purchased from Jiangsu Shengdi Pharmaceutical Co., Ltd. (Nanjing, China), 0.2 g/bottle, national standard H32020857. Fetal bovine serum (FBS) and DMEM-H medium were purchased from Gibco Company of the United States (Waltham, MA, USA). ALT, CRE, and UREA assay kits were obtained from Biosino Bio-Technology and Science Inc. (Beijing, China). Mouse IL-6, IL-10, IL-1β, and TGF-β ELISA kits were purchased from Beyotime (Shanghai, China).

Reference substances were used to compare the MS data and retention time (RT) of the identified compounds consisting of astragaloside IV, astragaloside III, Isoastragaloside II, Ononin, Calycosin-7-glucoside, 9-O-Methylnissolin 3-O-glucoside, Cycloastragenol, purchased from Chengdu Manster Biotechnology Co., Ltd. (Chengdu, China). For UPLC-MS analysis, LC/MS-grade acetonitrile, methanol, and formic acid were purchased from Thermo Fisher (Waltham, MA, USA), and water was purchased from Guangzhou Watsons Food & Beverage Co., Ltd. (Guangzhou, China).

### 3.3. Preparation of Plant Materials

#### 3.3.1. Preparation of Ethanolic Extract

After crushing the wild and cultivated Astragali Radix (AR) samples, 8–10 times their volume of 80% alcohol was added for hot reflux extraction. The extraction was performed 3 times, with each extraction lasting 2 h. The mixture was filtered while hot, and the combined filtrates were concentrated to dryness for later use.

#### 3.3.2. Preparation of Reference Standard

Reference substances were accurately taken and weighed. After adding methanol HPLC gradient grade to prepare 1 mg/mL standard solution, 50 μL single standard was mixed and diluted to 1 mL to obtain 7 standard mixed standards, which were stored in a refrigerator at 4 °C for use.

#### 3.3.3. Sample Preparation

The imitation wild AR and cultivated AR samples were powdered and sifted through a 50-mesh sieve. The extraction method: each powdered sample (1 g) was added into a 20 mL volumetric flask, 80% methanol was poured, sonication treatment was carried out for 30 min (250 W, 20 KHz), and the sample solution was filtered through a 0.22 µm membrane filter before injection into the UPLC-Q-TOF/MS system. Each group has six samples. The stability and repeatability of the methodology, which employed gradient elution, were determined by the repeat analysis of a pooled quality control (QC) sample, which was mixed with the 12 samples of AR. Finally, UPLC-Q-TOF/MS analysis was performed after proper dilution with methanol.

### 3.4. Pharmacological Experiments

#### 3.4.1. Cell Culture

4T1 breast cancer cells were purchased from the Cell Bank of the Culture Preservation Committee of the Chinese Academy of Sciences (Shanghai, China). 4T1 cells were cultured with DMEM-H containing 10% FBS and placed at 37 °C with 5% CO_2_ saturated humidity. Depending on cell metabolism, the cells were changed when they reached 80% growth, and 1:3 to 1:4 passages were performed 1–2 times a week.

#### 3.4.2. Animals

Female BALB/c mice (16.0–18.0 g) were purchased from SPF Biotechnology Co., Ltd., (Beijing, China). All animal experiments were approved by the Institutional Animal Care and Use Committee of IMPLAD (ethics approval number: SLXD-20210930016) and were carried out in agreement with the Guide for the Care and Use of Laboratory Animals (NIH publication #85–23, revised in 1985). All mice were housed under a 12-h light-dark cycle in a temperature and humidity-controlled room with free access to tap water and irradiated food. Modeling was started after one week of acclimatization and rearing.

#### 3.4.3. Mouse Grouping and Model Establishment

As shown in Figure S1, 60 healthy Balb/c female mice were selected for this experiment. 4T1 were inoculated subcutaneously on the right penultimate pair of mammary fat pads. Each mouse was inoculated with 100 μL cell suspension at a density of 5 × 10^6^/each. After 48 h of modeling, pink bumps were observed at the inoculation site to verify whether the modeling was successful. After one week of vaccination, the length (a) and width (b) of the tumor were measured with a caliper. Tumor volume was calculated as V = 1/2ab^2^. Mice with a tumor volume of 100 mm^3^ were selected for subsequent experiments.

In the experiment comparing the pharmacodynamic activity of two kinds of AR, mice were randomly divided into the following six groups (n = 6). The control group (CON) was treated with CMC-Na (i.g.) daily and then treated with vehicle (0.9% physiological saline, i.p.) once every 5 days. The CTX group was treated with CMC-Na (i.g.) daily and then treated with CTX (i.p.) once every 5 days, with a dose of 100 mg/kg [40]. The imitation wild and cultivated AR ethanolic extract (IAEE and CAEE) groups were treated with IAEE and CAEE (i.g.) daily, at a dose of 300 mg/kg, and then treated with vehicle (0.9% physiological saline, i.p.) once every 5 days. The AR ethanolic extract plus chemotherapy drug combination therapy groups (IAEE+CTX and CAEE+CTX) were treated with IAEE and CAEE (i.g.) daily, at a dose of 300 mg/kg, and then treated with CTX (i.p.) once every 5 days, at a dose of 100 mg/kg. The administration period was 16 days. During the experiment, the mice were free to eat and drink water.

#### 3.4.4. Determination of Tumor Volume, Mass, and Observation of Pathological Tissue

On days 14–29 of the experiment, the long diameter a (mm) and short diameter b (mm) of the tumor were measured daily with a vernier caliper, and the tumor volume (mm^3^) was calculated according to the formula V = 1/2ab^2^. The tumor growth curve was drawn according to the daily measured tumor volume to observe the effect on tumor growth after administration.

The mice were killed on day 30 after overnight fasting, mouse blood samples were collected for biochemical examination, and the tumors of each group were completely stripped and weighed. Tissue supernatants were collected for the evaluation of cytokines after centrifuging at 10,000 rpm for 10 min at 4 °C. The concentrations of IL-6, IL-10, IL-1β, and TGF-β were measured by ELISA kits. The harvested tumors of each group were retained in a volume of 1cm^3^, fixed in 4% paraformaldehyde, embedded in paraffin, cut into 5 μm sections, dewaxed and hydrated all tissue specimens, and stained with hematoxylin and eosin (H&E), and histopathological changes according to conventional procedures were observed.

#### 3.4.5. Evaluation of the Effect of Drugs on Main Organs

To detect whether the drugs involved in this experiment have potential toxicity to the main organs of the body, the increase or decrease in the relative body weight of the animals was monitored during the experiment, and the hunger weight was weighed on the 30th day of the experiment. The data were recorded, and the effect on the body weight of the mice after administration was observed. In addition, after the completion of the study on the efficacy of tumor inhibition, the blood of the mice was collected by eye picking, and the main organs were removed and fixed in 4% paraformaldehyde. The sections were stained with hematoxylin and eosin (H&E) staining. The serum of the mice was detected by an automatic biochemical analyzer for liver function, renal function, and other biochemical indicators, and histopathological analysis was performed according to the results of H&E staining.

### 3.5. UPLC-Q-TOF/MS Analysis

#### 3.5.1. UPLC-Q-TOF/MS Methods

UPLC separation was achieved on a Waters ACQUITY I-Class system (Waters Corporation, Milford, MA, USA) using a Waters ACQUITY BEH C_18_ column (2.1 mm × 100 mm, 1.7 μm, Waters Corporation, Milford, MA, USA). The column and autosampler were maintained at 30 and 10 °C, respectively. The flow rate was 0.3 mL/min, and the injection volume was 1 μL. The mobile phase consisted of 0.1% formic acid water (A) and acetonitrile (B), and the gradient conditions were as follows: 0–5 min, 10–20% B; 5–20 min, 20–65% B; 20–25 min, 65–90% B; 25–26 min, 90–10% B; 26–30 min, 10% B. The online UV spectra were recorded in the range of 200–400 nm.

Mass spectrometry was operated on the Waters Xevo G2-XS Q-TOF mass spectrometer (Waters Corporation, Milford, MA, USA) equipped with an electrospray ionization (ESI) source controlled by MassLynx 4.2 software (Waters, Corporation, Milford, MA, USA). A full scan was run in positive modes, with a mass range from *m*/*z* 100–1500 Da and with a 1 s scan time. Nitrogen was used as a nebulizer and auxiliary gas. In positive ion mode, the following parameters were found: capillary voltage of 3 kV; sampling cone voltage of 40 V; source temperature of 100 °C; desolvation temperature of 250 °C; cone gas flow of 50 L/h; desolvation gas flow of 600 L/h. The instrument was performed in both low-energy and high-energy scan functions, and the collision energy was 6 and 20–50 eV, respectively. Leucine enkephalin was used as a lock mass with a reference mass value at *m*/*z* = 556.2771.

#### 3.5.2. Construction of the Astragalus Compound Database

A local database of the genus *Astragalus* was created using Progenesis SDF Studio (https://www.nonlinear.com/progenesis/sdf-studio/, accessed on 13 February 2025). The compounds from *Astragalus* species were sorted and summarized by searching SciFinder, Pubmed, CNKI, and Google Scholar. By using Sci Finder to search for the compound’s name or structure as reported in the literature, confirm the single structure file of the chosen compound (.mol format), and then proceed with the transfer. All .mol files were consolidated into an internal database by Progenesis SDF Studio. The established database was used to identify the compounds.

#### 3.5.3. Data Processing and Analysis

The sample data collected by UPLC-Q-TOF/MS were imported into Progenesis QI 2.3 (Waters, Milford, MA, USA) software and ion forms such as [M+H]^+^, [M+Na]^+^, [M+K]^+^, [M+NH_4_]^+^, [2M+H]^+^, [2M+Na]^+^, [M+H-H_2_O]^+^, and [M+H-2H_2_O]^+^ were selected to unwrap the spectral data to improve the identification rate of the data. The data were imported in a .raw format, and then QI automatically selected a more standard sample for calibration based on the imported sample, followed by peak alignment, peak extraction, normalization, and compound identification based on the online database and the “*Astragalus* chemical composition database” constructed above. The measured compound information is compared and identified with the information in the reference substance and database. In addition, the compound abundance in the two Astragali Radix species is compared. SIMCA 14.1 was employed to process exported data, using principal component analysis (PCA) and orthogonal partial least-squares discriminant analysis (OPLS-DA), combined with an S-plot to analyze potential marker compounds. In S-plots, the significant differential retention time–exact mass (RT-EM) pairs were chosen and marked on the Progenesis QI for compound identification. The RT-EM pairs were then determined by variance analysis, with a *p*-value of ≤ 0.05 and a max fold change of ≥2.

## 4. Conclusions

This study investigates the differences in immune-enhancing activities between imitation wild and cultivated Astragali Radix. The findings demonstrate that imitation wild Astragali Radix exhibits significantly superior activity compared with its cultivated counterpart, offering a scientific basis for consumer preference for imitation wild Astragalus. Moreover, a comparative analysis of their chemical compositions identified biomarkers unique to imitation wild Astragali Radix. These biomarkers provide a foundation for the classification, grading, and quality control of imitation wild and cultivated Astragali Radix.

## Figures and Tables

**Table 1:** Compounds identified in the alcoholic extract of Astragali Radix by UPLC-Q-TOF/MS methodology. *: Indicates that the compound has a reference standard, ‘+’ indicates presence, ‘−’ indicates absence.

No.	Time (min)	Compound Name	Formula	Selected Ion	Experimental Mass (*m*/*z*)	Theoretical Mass (*m*/*z*)	ppm	IATE	CATE
1	3.30	*Rutin*	C_27_H_30_O_16_	[M+H]^+^	611.1623	611.1602	+3.5	+	+
2	3.44	3′,7,8-trihydroxy-4′-methoxyisoflavone	C_16_H_12_O_6_	[M+H]^+^	301.0718	301.0707	+3.7	+	+
3	3.69	Chrysoeriol-7-O-D-Glucopyranosyl-4′-O-α-L-rhamnopyranoside	C_28_H_32_O_15_	[M+H]^+^ [M+Na]^+^	609.1821 631.1619	609.1809 631.1639	+1.9 −3.2	+	+
4	4.67	Syringic acid	C_9_H_10_O_5_	[M+H-2H_2_O]^+^	163.0385	163.0391	−3.7	+	−
5	5.15	Quercetin 3-O-β-D-glucopyranoside	C_21_H_20_O_12_	[M+H]^+^	465.1031	465.1026	+1.1	+	+
6	5.33	Dihydroxy-6-methoxyaurone	C_16_H_12_O_5_	[M+H]^+^	285.0746	285.0759	−4.6	+	+
7 *	5.69	Calycosin-7-glucoside	C_22_H_22_O_10_	[M+H]^+^ [M+K]^+^	447.1302 485.0840	447.1284 485.0850	+4.0 −2.1	+	+
8	5.95	Verbascoside	C_29_H_36_O_15_	[M+H]^+^	625.2125	625.2121	+0.6	+	+
9	6.28	2′-Hydroxy-dimethoxyisoflavan-β-D-glucoside	C_23_H_28_O_9_	[M+H-2H_2_O]^+^	413.1596	413.1591	+1.3	+	−
10	6.69	Liquiritigenin	C_15_H_12_O_4_	[M+H]^+^	257.0807	257.0810	−1.2	+	+
11	6.85	8,2′-Dihydroxy-7,4′-dimethoxyisoflavan	C_17_H_18_O_5_	[M+Na]^+^	325.1046	325.1047	−0.4	+	+
12	7.10	Vanillin	C_8_H_8_O_3_	[M+H]^+^	153.0555	153.0549	+3.9	+	+
13	7.27	7-Hydroxy-2′, 3′, 4′, 5′-tetramethoxyisoflavan	C_19_H_22_O_6_	[M+H-2H_2_O]^+^	311.1270	311.1276	−1.9	+	+
14	7.44	Astraflavonoid B	C_27_H_36_O_19_	[M+NH_4_]^+^	682.2176	682.2195	−2.8	+	+
15	7.62	Rhamnocitrin-3-O-β-D-glucopyranoside	C_22_H_22_O_11_	[M+Na]^+^	485.1065	485.1060	+1.0	+	+
16	7.69	Oroxylin A	C_16_H_12_O_5_	[M+Na]^+^	307.0587	307.0582	+1.6	+	+
17 *	8.25	Ononin	C_22_H_22_O_9_	[M+Na]^+^	453.1161	453.1162	−0.2	+	+
18	8.64	Homobutein	C_16_H_14_O_5_	[M+H]^+^	287.0910	287.0919	−3.1	+	+
19 *	8.68	9-O-Methylnissolin 3-O-glucoside	C_23_H_26_O_10_	[M+H]^+^	485.1426	485.1416	+2.1	+	+
20	8.80	Oxytropisoflavan B	C_17_H_20_O_6_	[M+H]^+^	321.1343	321.1338	+1.6	+	+
21	8.93	Astragaloside VIII methyl ester	C_48_H_78_O_17_	[M+NH_4_]^+^	944.5591	944.5583	+0.8	−	+
22	9.17	Rhamnocitrin	C_16_H_12_O_6_	[M+H]^+^	301.0721	301.0712	+3.0	+	+
23	9.40	Astroolesaponins F	C_49_H_78_O_18_	[M+H]^+^	955.5261	955.5261	−0.5	−	+
24	9.52	AstragalosideII	C_43_H_70_O_15_	[M+Na]^+^	849.4634	849.4612	+2.6	+	+
25	9.81	3,5-Dicaffeoylquinic acid	C_25_H_24_O_12_	[M+H]^+^	517.1359	517.1346	+2.5	+	+
26	10.78	Betulinic acid	C_30_H_48_O_3_	[M+H]^+^	457.3672	457.3682	−2.2	+	+
27 *	11.05	Astragaloside III	C_41_H_68_O_14_	[M+H-H_2_O]^+^	767.4568	767.4582	−1.8	+	+
28	11.17	Astroolesaponins B	C_48_H_78_O_19_	[M+H]^+^	959.5210	959.5216	−0.6	+	+
29 *	11.53	Astragaloside Ⅳ	C_41_H_68_O_14_	[M+H]^+^	785.4667	785.4887	−2.5	+	+
30	11.63	Robinioside B	C_48_H_76_O_20_	[M+H]^+^	973.5021	973.5008	+1.3	+	+
31	11.85	Robinioside F	C_48_H_78_O_19_	[M+H]^+^	959.5216	959.5210	+0.6	+	+
32	12.17	Chrysoeriol-4′-O-α-L-rhamnopyranoside	C_22_H_22_O_10_	[M+H]^+^	447.1302	447.1291	+2.5	+	+
33	12.24	Astraisoolesaponins A1	C_48_H_72_O_21_	[M+H]^+^	985.4636	985.4644	−0.8	+	+
34 *	12.56	Isoastragaloside II	C_43_H_70_O_15_	[M+H]^+^	827.4780	827.4793	−1.6	+	+
35	12.73	Cloversaponin III	C_42_H_64_O_16_	[M+H]^+^	825.4252	825.4273	−2.5	+	+
36	13.10	Cyclocephaloside II	C_43_H_70_O_15_	[M+H]^+^	827.4780	827.4793	−1.6	+	+
37	13.29	Kaempferol-4′-methyl ether	C_16_H_12_O_6_	[M+H]^+^	301.0721	301.0712	+3.0	+	+
38	13.34	AstragalosideI	C_45_H_72_O_16_	[M+H]^+^	869.4914	869.4899	+1.7	+	+
39	13.75	IsoastragalosideI	C_45_H_72_O_16_	[M+H]^+^	869.4914	869.4899	+1.7	+	+
40 *	14.79	Cycloastragenol	C_30_H_50_O_5_	[M+H-2H_2_O]^+^	455.3531	455.3525	+1.3	+	+
41	14.96	2′,4,4′-trihydroxychalcone	C_15_H_12_O_4_	[M+H]^+^	257.0826	257.0814	+4.7	+	+
42	15.02	Astroolesaponins A	C_48_H_76_O_18_	[M+Na]^+^	964.5053	964.5008	+4.7	+	+
43	15.48	Astroolesaponins D	C_48_H_74_O_19_	[M+H]^+^	955.4944	955.4903	+4.3	+	−
44	16.82	Astragaisoflavan D	C_34_H_34_O_10_	[M+H]^+^ [M+Na]^+^	603.2254 641.1813	603.2230 641.1789	−4.5 +3.7	+	+
45	16.91	AcetylastragalosideI	C_47_H_74_O_17_	[M+H]^+^ [M+Na]^+^	911.5010 933.4869	911.5004 933.4824	+0.7 +4.8	+	+
46	20.08	Methylnissolin	C_17_H_16_O_5_	[2M+H]^+^	601.2070	601.2074	−0.7	+	+
47	21.51	Swainsonine	C_8_H_15_NO_3_	[2M+H]^+^	347.2173	347.2182	−2.6	+	+
48	22.42	Stigmasten-4-en-3-one	C_29_H_46_O	[M+H-H_2_O]^+^	393.3535	393.3521	+3.6	+	+

**Table 2:** Different chemical constituents of Astragalus membranaceus by different cultivation methods.

No.	Min *m*/*z* (n)	Compound Type	Compound Name	VIP
1	5.69_446.1226n	Isoflavones	Calycosin-7-glucoside	9.40
2	7.62_462.1173n	Flavonoids	Rhamnocitrin-3-O-β-D-glucopyranoside	3.35
3	8.25_430.1276n	Isoflavones	Ononin	6.31
4	9.81_516.1277n	Organic acids	3,5-Dicaffeoylquinic acid	7.82
5	16.91_910.4919n	Saponins	Acetylastragaloside I	3.01

## Data Availability

All experimental data and detailed experimental procedures are available upon request from the corresponding authors.

## Supplementary Materials

The following supporting information can be downloaded at https://www.mdpi.com/article/10.3390/molecules30040923/s1, Figure S1: Example experimental chart comparing the antitumor efficacy of IAEE and CAEE. Figure S2: Overlap pattern of QC sample positive ion mode TIC.
