# Supplementary figures and images for: Comparison of the Immune Enhancing Activity and Chemical Constituents Between Imitation Wild and Cultivated Astragali Radix
Source: Molecules. 2025 Feb 17;30(4):923. doi: 10.3390/molecules30040923 (PMC11858062; doi:10.3390/molecules30040923)

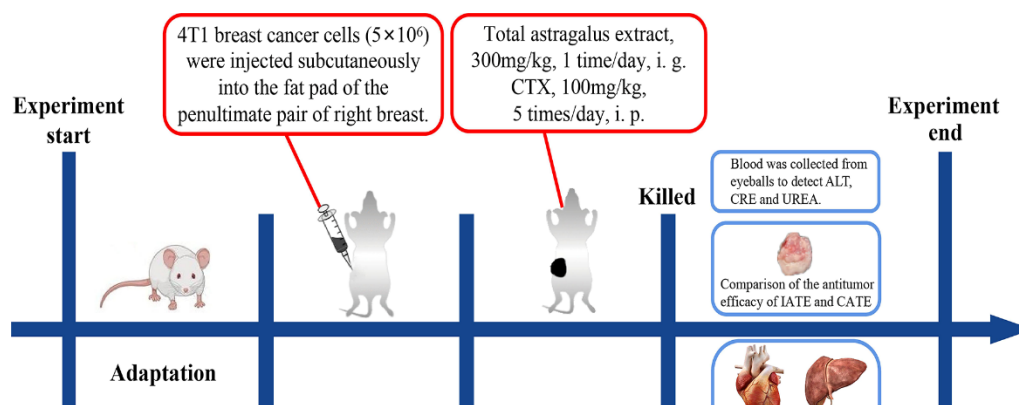

**Figure S1** Example experimental chart comparing the antitumor efficacy of IAE and CAEE

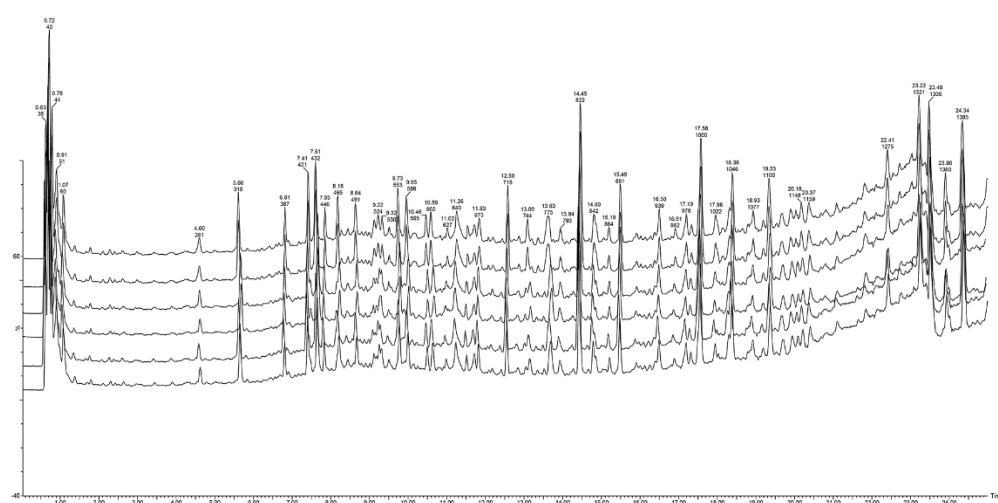

Supplement: Supplementary file 1 [file molecules-30-00923-s001.zip › molecules-3441866-supplementary.pdf]
